# When the group practice breaks up: a qualitative study

**DOI:** 10.1186/1471-2296-14-53

**Published:** 2013-05-03

**Authors:** François Marechal, Dorothée Schmidt, Evelyne Lasserre, Laurent Letrilliart

**Affiliations:** 1 Département de médecine générale, Université Claude Bernard Lyon 1, 8 avenue Rockefeller, Lyon Cedex 08 69373, France; 2 S2HEP, Université Claude Bernard Lyon 1 & ENS Lyon 43, Boulevard du 11 Novembre 1918, Villeurbanne Cedex 69373, France

**Keywords:** Practice breakup, Group practice, Partnership, General practice, Suffering at work, Contract

## Abstract

**Background:**

Group practices are increasingly common for primary care physicians worldwide. Although breakups are likely to happen frequently within group practices, their process has not been studied to date. The aims of this study were therefore to explore the reasons for breakups of group practices of general practitioners and to describe the associated feelings.

**Methods:**

We conducted a qualitative study consisting of in-depth interviews of 21 general practitioners and one secretary from past group practices in the Rhône-Alpes region, France, who experienced a breakup.

**Results:**

When getting started in group practice for the first time, young doctors did not feel ready and supported, and did not necessarily share the same expectations as their partners. The reasons for the breakups involved imbalances within the groups, contrasting working and management styles, and breakdowns in communication. The breakup process often generated long-persistent feelings of suffering and failure for almost every partner who experienced a breakup, particularly for the partner who was leaving.

**Conclusions:**

Weakening factors exist from the very beginning of a partnership, and problems are likely to increase at every change or event occurring in the group. We provide several recommendations, including fair management, a shared project based on a precise contract, the consultation of third parties as necessary and, in the worst case scenario, leaving the group practice in time.

## Background

Group practices are increasingly common for general practitioners (GPs) and family physicians worldwide. Private group practice has become the predominant mode of primary medical care practice in many countries, such as Australia, Canada, Denmark, the Netherlands, New Zealand, the Slovak Republic or the UK [1]. The proportion of group practices can vary widely, from less than 20% in Italy to more than 90% in the UK and Canada [2]. In France, this proportion was estimated at 54% in 2009 and incentives are being implemented to facilitate group practice in areas where there is a shortage of doctors [3]. Private practice is also the predominant type of general practice in France. In group as in solo private practices, the activity of French GPs is paid on an individual practices, the activity of French GPs is paid on an individual fee-for-service basis and income is generally not shared between partners. For some authors, this system is perceived as a barrier for developing cooperation in group practices [4].

Working together indeed offers several potential advantages, such as improved quality of life for the practitioner, increased continuity of care for the patient and better sharing of experience between healthcare professionals [5]. Actually, GPs working in group practices more frequently declare that they work fewer days per week but dispense more medical consultations per day than GPs working in solo practice. Group practice can free up the physician’s time for training or supervising students [3]. It may even improve quality of care, through longer consultations [6,7]. A few studies have shown that working in a group practice is also demanding, as building and maintaining a strong, supportive partnership requires time and effort [8]. Teamwork produces tensions, especially over the distribution of the workload and communication with colleagues [9]. These tensions can be perceived by the patient and negatively impact the quality of care. In the UK, a number of newly appointed partners had previously worked in another group practice [10]. In France, about ten percent of GPs left their group practice at least partly because of conflicts with partners [11].

To our knowledge, the breakup process within group practices has not been studied to date. The aims of this study, entitled “ICARE MG”, were therefore firstly to explore the reasons for breakups in group practices and secondly to describe the practitioners’ feelings about this experience.

## Methods

The study was based on in-depth interviews, conducted in accordance with the grounded theory [12].

### Sampling

A purposeful sample of GPs who had been confronted with a breakup in a group practice was selected. Chosen from physicians in the Rhône-Alpes region of central France, the sample brought together male and female practitioners of various ages, group practice sizes and locations. A first sample was built by advertising in the journal of the Medical Council of the Rhône county and by sending e-mails to members of the local general practice teaching faculty. Using the snowball technique, we also asked the participants from the first sample to inform us of any other GPs they knew outside of their own group practice that had experienced a group practice breakup.

Twenty-two interviews were conducted, eleven for each investigator, at the interviewed physicians' offices. The first physician interviewed in each group served as the index and we tried to retrace the breakup of their own group practice by interviewing all of their partners, including the assistant if she had worked at the practice for several years. We therefore asked the index physicians for permission to interview their previous partners and how to contact them. There were 10 people we did not manage to interview: 3 refused, 3 could not be contacted and 4 were members of a group whose interviewed physician asked us not to include his 4 former partners in the study because he feared they might harm him.

### Data collection

A semi-structured topic guide based on a bibliographic review and discussion between the authors was developed. It included open-ended questions concerning the circumstances of the partnership setup, the organisation and rules for working together, the reasons for the breakup, and the lessons learnt from this experience. The guide was modified based on the data that had been collected from the first interviews. Data from each interview was audio taped with the participants’ consent. Information provided by partners was never disclosed to other partners by the interviewers. The interviews lasted 35 to 85 minutes.

### Data analysis

Interviews were transcribed verbatim anonymously and analyzed using QSR International's NVivo 9 software [13]. Content analysis was based on constant comparisons, allowing emergent themes to be identified. Regular review and discussion of evolving themes between the authors supported data interpretation. As per French regulations, ethical approval was not required for this study. However, it was covered by a general agreement obtained from the French Data Protection Authority (CNIL, record number 1089806).

## Results

Nine group practices, twenty-one practitioners and one secretary were included in the study (Table 1). The group practices comprised from two to seven practitioners. Of these practitioners, there were six females and fifteen males aged 38 to 77. Among these group practices, two were composed of women only, four of men only and three were mixed. The time between entering the group practice and the breakup ranged from two to twenty years.

**Table 1:** Characteristics of group practices and breakups

	**Group practice**			**Breakup**		
**Group**	**Number of physicians**^**1,2**^	**Type of contract**	**Area**	**Duration before breakup (yrs)**	**Main reasons**	**Involvement of lawyers**
A	3 (A1,A2,**A3**)	SCM	Urban	5	Difficulty in welcoming a new partner Breakdown in communication Financial disagreements	Yes (Trial)
B	4 (B1,B2,**B3**,B4,B5^3^)	SCP^4^	Semi-rural	3	Incompatible style and personality Leadership conflict	Yes
C	3 (C1,C2,***C3***)	SCP	Semi-rural	19	Family problems Altered quality of life Heavy workload Breakdown in communication	No
D	3 (D1,D2,***D3***)	SCM	Urban	6	Incompatible style Family intrusion Breakdown in communication Difficulty in welcoming a new partner	Yes
E	2 (**E1**,E2)	SCM	Rural	5	Incompatible style Unsuitable office (loud and dark) Unachieved project of medical home	No
F	5 (**F1**,*F2*,*F3*,*F4*,*F5*)	SCP	Rural	2	Incompatible style Feeling of persecution Difficulty in welcoming a new partner	Yes
G	7 in 2 surgeries (G1,G2,G3,**G4**,*G5*,*G6*,*G7*)	SCM	Urban	2	Incompatible style Difficulty in welcoming a new partner Financial disagreements	No
H	2 (**H1**,*H2*)	SDF	Urban	5	Difficulty in welcoming a new partner Incompatible style and personality Breakdown in communication	No
I	2 (I1,**I2**)	SDF	Urban	20	Distrust Poor management of the practice due to illness	No

SCM (Société Civile de Moyens) = non-trading company (sharing of costs). SCP (Société Civile Professionelle) = professional limited liability partnership (sharing of responsibilities, fees and costs). SDF (Société de fait) = non substantifically juridical company (sharing of costs).

^1^Identifiers in bold characters denote the partners who left first in each group. ^2^Identifiers in italics denotes the partners who were not interviewed. ^3^Secretary. ^4^With distribution of fees based on activity.

Four major themes were identified from the analysis, from the creation of the group to the lessons learned from the breakup experience, including the reasons for the breakup and the feelings associated with this experience.

### The process of entering a group practice

Setting up in a private practice, even in a group practice, was perceived as risky, mainly for financial reasons. Thus, young doctors did not feel ready for the challenge and expected support from colleagues who were not always eager to help. The interviewees usually joined a group practice very quickly, often seizing an opportunity. They were not very careful and sometimes had not worked at the group practice, even for a short period, before becoming a partner. For instance, the length of time the physicians had worked as a locum at the group practice prior to joining it varied from 0 to 3 years. When joining a practice, most of the practitioners had no apprehension towards the group, while a few immediately feared possible incompatibility within the group practice. Some of them accepted to join a practice located in a place where they did not want to live in, simply to avoid an area with a high density of GPs.

G4: “I quickly decided to set up in a place I didn’t know, where I had never worked as a locum.”

B3: “When I joined the practice, I was asked very precise questions, such as “What kind of newspaper do you read?” (…) If I’d answered “Le Figaro” (a French right-wing newspaper), I’m not sure I would have been accepted by the group!”

Some practitioners joined a group practice for financial reasons, for example in order to reach a sufficient level of activity quickly within the group or to share the costs of equipment and a secretary. Others highlighted their wish to organise their practice, in order to reconcile their private and professional lives. Another expected benefit was team spirit, based on sharing of the workload, ideas and knowledge. Sometimes, the expectations were excessively high, given the actual conditions of practice, especially when the practitioner had not worked as a locum within the group practice before.

B2:“I thought that, in principle, since they were apparently playing by the rules, I would have work right away, which was in fact my biggest concern.”

B4 “A group practice seemed to me to be somewhat of an ideal. On many levels, such as duties, working hours, secretarial tasks, shared areas, never feeling lonely, etc.”

F1: “I had big illusions, and really high expectations concerning joining a group practice.”

### The reasons for the breakup

The main imbalance laid in the uneven distribution of medical activity, which particularly affected new partners. They reproached their associates for not having helped them to develop a high enough level of activity. Some of them even reported that they left the group because they did not earn a sufficient amount of money for living. The notion that certain patients “belonged” to a doctor generated a competitive climate. Several partners found that the sharing of the costs of the group practice was unfair or that common duties were unequally distributed. While the partners wanted to be acknowledged by their associates, a kind of hierarchy often developed, especially with new partners, based on seniority or a higher level of medical activity. At worst, a partner felt dominated, manipulated or even persecuted by another perceived as powerful, particularly if nobody in the practice dared to contradict him. These imbalances sometimes generated a survival instinct in one or more of the partners.

G4: “He [G3] used to see between 35 and 45 people a day. When I started, I used to see 6! (…) They didn’t help me. They even tried to make it worse by coming and getting my patients in my waiting room and saying: “Umm, I can see you quicker!”

G2: "Since he was working less, he was always complaining because he had to give as much as we did for the running of the practice.”

Incompatible personalities were also the cause of tensions within the group practices. Extremely different working habits, concerning for example usual length of consultations or participation in continuing medical education, were specifically difficult to deal with. They were sometimes related to differing conceptions of quality of care, and could lead to intolerance. The management of the practice led to financial or organisational disagreements. New partners particularly expected to organise the practice as closely as possible to their ideal, even by trying to change their partners' habits. The intrusion of personal issues into the practice was in some cases the main reason for a breakup.

B4 – “We had arguments when he [B3] could no longer accept the fact that we didn’t try to imitate him.”

F1: “When I arrived (…) I wondered: “Wait a minute, there’s a place and there’s no meeting. Should I do something about it?” I said to myself, “Well, I’m going to change things” and so I suggested having a meeting. (…) In fact, it never worked. Every time, there was only one or two of us …”

D1: “We had to take on board the fact that our third colleague [D3], at a certain point in time, was no longer able to distinguish between his professional life and the problems in his personal life.”

Lack of communication or misunderstandings between partners were consistently reported at one stage or another in the breakup process and were sometimes fostered by workaholism. For example, it could happen that one partner was speaking about interpersonal relationships whereas another was speaking about purely legal issues. When trust was lost, it appeared difficult to regain, especially in the event of a breach of professional ethics. The conflict worsened if the partners did not question themselves individually. The secretary interviewed gave a precise analysis of the group practice she had worked at for thirty-seven years.

B1: “Whenever there was a big crisis within the group, it was when we no longer took the time to communicate.”

A3: “It also helped me realise that… there are some people who don’t care… who don’t have any ethics, who are not honest and who are ready to steal patients from their colleagues.”

B4: “The oldest member [B3] found himself alone with his idea of medicine, his idea of the group, and he wasn’t flexible or open enough to accept the fact that there might be other ideas, other ways of doing things, etc.”

### The associated feelings

The breakup process was described as extremely painful, stressful and exhausting, particularly for the partner who was leaving, and in a few cases was even a cause of illness. These feelings were sometimes related to a humiliating relationship, where a "naive" partner faced one or more "malicious" partners, or when patients changed doctors. This suffering was so invasive that it could lead to depression, even if the partner did not necessarily realise it at that time. Indeed, this illness was sometimes revealed by family, friends or even patients. In one practice, some of the physicians even reported that several former partners had committed suicide. Feelings were so hard before the breakup that some of the physicians described a form of relief just after the breakup or when they discovered new professional horizons. Distrust was so intense that some physicians were described as "paranoid" by their partners. Resentment against partners was in some cases accompanied by anger and a loss of self-control. The psychological pain was often compared to that caused by a divorce. It could be associated with the fear of reprisals or of another breakup in a new partnership. For most physicians, that pain was still present at the time of our interview, sometimes decades after the breakup.

I2: “And the fact that my partnership with I1 fell apart was so distressing for me, it was such an upheaval (…) it was the only thing I could think of, the only thing I could see. I was talking about it all the time, I was completely overrun by it.”

A1: “There were a certain number of patients who changed from me to her. (…) I knew very well it was going to be like that but, for some of them, I probably didn’t measure the pain it was going to cause. The bastards!”

B4: “Over several years, B3 had become increasingly paranoid about the three of us.”

D2: “I would say that it’s a bit like after a divorce. You need to leave yourself a bit of time! You try to heal the wound, as it were.”

F1: “It’s one of the biggest traumas of my life. On a scale of 1 to 10, I would give it an 8. It’s a trauma I have transformed into something positive.”

G4: “For me, it is over. But apparently it isn’t really over [laughs] (…) I put a big lid on top of it and I don’t want to hear about it again. (…) It still troubles me today [in a very emotional voice].”

Physicians who experienced a harsh breakup usually felt disappointed and defeated, and tended to devalue themselves. They expressed regrets, particularly regarding the contract. The feelings of suffering and failure were sometimes associated with feelings of betrayal by their partners. In some cases, this extended out of the practice, to disillusion with all interpersonal relationships. The shock wave often impacted their future activity, with physicians deciding to work in a solo practice or be employed as a GP, to specialize in psychiatry or in skincare, or even to stop working altogether. In some cases, it also affected their personal life, leading to divorce.

G4: “I felt completely worthless. (…) Even so, objectively, for me, it’s a real acknowledgement of failure.”

A2: “(What makes me bitter is) having become disillusioned with humankind. And ever since, I don’t believe in anything anymore, I don’t believe… you see? That anyone is reliable.”

The litigious temperament of some partners and the eventual involvement in a lawsuit made a breakup even more difficult to face. Four of the nine breakups have actually been managed by lawyers, one breakup leading to a trial with an appeal seven years later. The breakup experience was further complicated by the perceived incompetence of professionals involved, including notaries, accountants, lawyers and representatives of the General Medical Council. Loneliness in the practice contributed to harsh feelings and was exacerbated by the lack of communication. Resentment ran higher at practices where partners no longer respected each other. We noted that an old friendship usually did not survive a professional breakup and even worsened this experience. On the contrary, an understanding spouse was a good support for several of the physicians interviewed, who reported that their wife had helped them to analyze the situation and their own feelings. A change of job or new professional prospects helped physicians to deal with the breakup, which could then proceed peacefully. However, partners remaining in the practice, especially in larger ones, were usually less affected than their partner who left and had to find another practice. One partner was so detached from his past experience that he did not remember the name of his former partner during the interview.

A3: “At the end, we even resorted to communicating by certified mail, before I left!”

D2: “We had to call upon lawyers to sort out the problem. (…) And it still isn’t finished! (…) There’s going to be more fighting.”

D2: “The male partner [D3], yes, he was a friend: we’d known each other for 20 years, we ate lunch at each other’s houses… Anyway! We’d gone on holiday together, you see… There we are! (…) Now, we’re at each other’s throats! I would say that he’s someone I don’t like at all! Not to say worse.”

G4: “One day my wife said to me, “You have to get it together, you have to do something, because you’re becoming as much of an ass as the others (the other partners).”

G3: “It (the breakup) happened without any particular problem.”

### Lessons learnt from the experience

To build unity within the group, some physicians suggested sharing a project with common objectives. Partners should be selected with care, based on similar personalities and professional styles, after a probationary period. It was specified that working together on a shared project required some basic human qualities such as honesty, respect, tolerance and open-mindedness. Making concessions and accepting differences as complementary was considered invaluable, as in a marriage. Constant questioning of oneself and taking special care of relationships with the partners was recommended in order to make a partnership last while allowing for change and evolution within the group.

G4: “You have to require a probationary period and a right to rescind, which should be at least 6 months in my opinion, no less.”

G3: “It’s just like a couple (…) You have to make concessions.”

A1: “ It’s never over! (…) It’s an everyday task. (…) You have to be careful of the others.”

D2: “Inevitably, one day or the other, if you have different medical practices, you’ll inevitably have different goals and after some time, to set your goals and start working towards them, the logistics won’t be the same. So there will be dissensions concerning the logistics. And, if there are disagreements about logistics, there are disagreements about financial aspects, and once you start talking money… it becomes very sensitive.”

Times for meetings should be scheduled and adhered to. Discussion between partners should occur in a neutral, convivial environment and be clear enough to avoid any kind of misunderstanding. Physicians should also spend specific times together, even without practice-related problems to solve. The physicians interviewed suggested fair, concerted sharing of common duties, workload, space and secretarial time. They recommended that no partner should have excessive power or authority over the others. Personal and professional lives should be separated. In particular, some physicians insisted that spouses should not interfere in the practice. Others suggested having cordial but not overly friendly relationships with partners.

B5: “The meetings were very spread out, but we felt better after a meeting. We felt the pressure rise before a meeting, then there was the meeting and it went down again, in fact.”

The main suggestion was to anticipate and to state clear internal rules for the management of the practice in the contract, including clauses for a potential breakup. The group should seek assistance from competent, motivated professionals such as notaries, accountants, lawyers or representatives of the General Medical Council. The physicians considered it was important to comply with the notification period before a breakup.

B4: “There have to be staff rules which are also precise, so you know how to manage holidays, how to manage absences and how to manage practice duties, if you have decided to set some up.”

A3: “The contract is not for managing things that run smoothly. It’s for planning ahead when things won’t work.”

B1: “Planning for the separation is extremely important!”

## Discussion

Most general practice breakups were the result of loneliness and suffering at work, which were often present long before the breakup and tended to increase over time. The breakup process was rarely smooth, illustrating that partnerships, like marriages, can be in hell rather than in heaven [14]. Most of the GPs who left group practices were very satisfied afterwards and felt that the transition had been successful, which is consistent with findings reported in England [9]. However, the feeling of failure persisted sometimes for years after the breakup, especially in case of a lawsuit and in the event of a trial. Globally, physicians felt they received little or poor support throughout the entire breakup period.

### Weakening factors in group practices

Life in group practices is punctuated by risky steps. Weakening factors exist from the very beginning of the partnership, and problems are likely to increase at every change or event occurring in the group.

The physicians interviewed had not been trained at GP practices during their medical studies and had not been taught how to manage a contract. Moreover, they usually only had little experience as a locum, as observed also in other countries, even if the locum time has been increasing in the recent years [15,16]. They often neglected to discuss certain important issues with their partners about the contract, particularly the breakup clauses. Their lack of experience led them to have unrealistic expectations, which contributed to their disappointment when they discovered the actual conditions of practice. Long-standing partners often had no experience in welcoming a new partner. They acted as if they expected new partners to accept their conditions and to adapt to their existing organisation, without imagining they might have to make any special efforts or accept any possible constraints for themselves.

Differences in style can affect the concept of partnership [17]. Personality, schedules, medical activity and relationships with patients form part of each physician's working style, which can vary widely from one doctor to another. Physicians naturally consider their own style as the best and are tempted to impose it on their partners. Their working styles are influenced by their values [18]. For instance, length of consultations or availability to patients are easily associated with quality of care [9]. Conflicting styles and values, combined with a lack of discussion or tolerance, can lead to unbalanced relationships and splitting partners into groups, especially when partners share patients. Trust, which is the foundation of any successful collaboration and a prerequisite for good quality care [9], can consequently be broken, leading to conflict every time a decision has to be made [14,19].

Every change in the group raises the possibility of confrontation and exacerbates pre-existing tensions. In particular, the arrival of a new partner can alter the previous balance of personalities within the group [20]. Lack of discussion at this time can be detrimental to the group and lead to the beginnings of a breakup.

### Influence of healthcare organisation

Some physicians decide to set up private practices in order to be their own boss, while others have no alternatives. In this private model, young physicians are typically vulnerable because they earn virtually nothing for months before making a profit. This competitive system reinforces individualism, which is also grounded in the law that makes each physician responsible for their own medical procedures [21]. As opposed to team spirit, individualism sometimes supplants relationships and personal investment in the group. This can, for instance, lead each partner to the unpleasant feeling that they have more duties in the practice than the others. The difficulty lies in holding meetings regularly over time despite each partner’s workload [5,8]. In a salaried system, team meetings are likely to occur when they are organised by a manager, but in return the physicians have no opportunity to choose their colleagues. To our knowledge, no study has focused on team dysfunctions in this type of healthcare system.

In the French system, private practices require strong involvement from GPs, and a breakup means a great personal failure to them. Furthermore, they do not get any administrative, logistical or even medical support. These difficulties may worsen in the future, due to the planned development of many multi-professional medical centers in France, which bring together healthcare providers from various professional cultures [22]. A surveillance system would therefore be useful to monitor the frequency of breakups.

### A generation gap

The change in medical demography is partly responsible for differences in styles and values between generations of physicians. In broad terms, senior doctors are used to making themselves more available for their patients, fearing to lose them, while junior doctors are more interested in organizing their time schedule and maintaining their quality of life [5]. Competitiveness can be so deeply rooted in older doctors' habits that, even when their activity has reached saturation, they sometimes endlessly continue to take on new patients.

Moreover, older doctors tend to reproduce a hierarchical organisation, in which some privileges are given to senior partners [23]. According to the concept of "initiation by disadvantage", new partners are often given all of the thankless duties or asked to contribute as much as their partners to the practice, even though their level of activity is lower [17]. Older doctors then act as if they did not have any obligation towards their partners, unlike towards their patients, to whom they are usually fully devoted. Most of the time, medical students are trained in hierarchical hospital settings and may have difficulty adapting to the more horizontal model of private group practices. Nowadays, the development of training courses in private practices at both pregraduate and postgraduate levels should better prepare them for partnership work [24].

### Strengths and limitations of the study

Due to medical demography, the sex ratio of the physicians interviewed was unbalanced (2.5 males to 1 female). However, the purposeful sampling process we used, in order to include women, allowed for the closest possible matching with the current trend in feminisation of the profession. Actually, we could not identify any gender specificity in the reasons for, or in the feelings associated with breakups. The high median age of the physicians (57 years) can be explained by their late start in the profession and the length of time between the breakups and our interviews. The proportion of clinical trainers was higher in our sample (7 GPs out of 21) than in the Rhône-Alpes region (9%) [25]. But trainers and non-trainers were often mixed in group practices, and this status did not prove to be involved in the breakup process. The range of group sizes in our sample, which predominantly included groups of two or three partners, was close to the range of sizes of French group practices in 2009 (67% versus 77%) [3]. We cannot exclude that missing or refusing partners may hide important information, especially in the two groups where only one partner was interviewed. However, data saturation was reached, as no new reason for breakup emerged after a dozen of interviews. Because of the time lag between the breakups and the interviews (median: 12 years), a memory bias, especially on the reasons for the breakups, cannot be excluded. However, the in-depth interviews should have limited this risk. Conversely, this time lag highlighted the long-term suffering of physicians, which may explain most of the refusals to be interviewed. Indeed, some physicians were reluctant to participate in this study simply because it was so hard for them to recall their trauma, even twenty years after. One physician, fearing reprisals, did not allow us to interview his former partners, despite the guarantee of anonymity. Paradoxically, for those reluctant physicians, the interview probably acted as a sort of psychotherapy, since they could not stop talking.

### Some recommendations

Because the skills and resources to create a supportive working environment all come down to fair, equitable collective management [20], an equal distribution of common duties is the first step to balancing a group. A rotation of duties could be established, with each partner alternately being manager, accountant or in charge of purchasing equipment [14]. At larger practices, an appointed mediator could focus on interpersonal problems or conflicts. At smaller practices, a team management consultant would be more appropriate, provided that they are not too expensive or are offered by a professional organisation such as a General Medical Council [14]. With a duty rotation, each partner deals with difficult or menial tasks and no one cumulates too many responsibilities [19]. Nevertheless, it seems reasonable to lighten a new partner's duties for a smoother integration, while orientating new patients towards him. More senior partners could potentially act as mentors for new partners in their first months at the group practice [14]. Efficient communication is a prerequisite for a successful partnership, by making room for every partner's concerns and by defusing tensions [26]. If possible, there should be a meeting room in the practice and specific time provided for formal and informal meetings [8,9]. Memos are sufficient for simple information, but meetings are necessary for complex or sensitive issues [19].

A new partnership contract should be discussed in detail and customised for each new member. It forces the new partner to clarify his expectations and fears, and their partners to update their own, which can vary with age and personality [5]. Topics such as sharing of costs or level of professional commitment need to be discussed in the contract. A probationary period, of at least several months, seems advisable [27]. Entering a group practice should not necessarily mean committing until retirement. A potentially renewable fixed-term contract could be established to help any partner to get started. Determining shared objectives at the beginning of a group practice federates its members into a team [28].

Using a third party may be useful at various stages for a group practice. In France, the General Medical Council provides standard contracts for partnership, but does not offer customised agreements for special circumstances. Calling upon lawyers or notaries to establish a contract with clear rules could later prove useful, especially in the event of a breakup. An internal or external assessment of the organisation of the practice could also help to improve its management, as with the Work Relationship Assessment Form, which was specifically created for group practices in the USA [19]. Secretaries are often the first witnesses to conflicts, and their views should be especially listened to [14]. When looking for a new partner, a third party such as a recruitment firm or head-hunter could help to hire the best possible candidate to ensure the group's balance while energizing it [20]. However, the feasibility of such external consulting is worth considering, from both an economic and cultural point of view, as medicine is a business unlike any other. Emotional support from trusted persons is also important before and after a breakup.

Sometimes the breakup can become unavoidable or preferable, to ease the suffering and prevent festering of relationships, despite a moral obligation towards partners or patients. In such situation, relationships with partners should be rational rather than based only on emotions, and each physician should remain respectful to his or her partners, during the partnership as well as during the breakup process. However, developing new professional prospects, starting a new partnership and building a new list of patients can be difficult for a physician weakened by a breakup [9,29].

## Conclusions

Breakups are mostly favored by imbalances between partners, particularly in medical activity, by incompatible personalities or working habits, and by a lack of communication within a group practice. These weakening factors exist from the very beginning of a partnership, and problems are likely to increase at every change or event occurring in the group, which can lead to a painful breakup. Competition present in fee-for-service healthcare systems may favor this process. Some recommendations on how to avoid breakups or to attenuate the related painful feelings are provided. They include fair management, a shared project based on a precise contract, the consultation of third parties as necessary and, in the worst case scenario, leaving the group practice in time.

## Competing interests

The authors declare that they have no competing interest.

## Authors’ contribution

FM and DS collected the data. FM, DS, EL and LL collectively analyzed the data. FM, DS and LL wrote the manuscript, which was reviewed by EL. All authors approved the final manuscript.

## Pre-publication history

The pre-publication history for this paper can be accessed here:

http://www.biomedcentral.com/1471-2296/14/53/prepub
